# *In vivo* Effects in Melanoma of ROCK Inhibition-Induced FasL Overexpression

**DOI:** 10.3389/fonc.2015.00156

**Published:** 2015-07-14

**Authors:** Iotefa Teiti, Bertrand Florie, Christine Pich, Rémi Gence, Isabelle Lajoie-Mazenc, Philippe Rochaix, Gilles Favre, Anne-Françoise Tilkin-Mariamé

**Affiliations:** ^1^Unité INSERM UMR 1037, CRCT, Toulouse, France; ^2^Université Paul Sabatier, Toulouse, France; ^3^Institut Universitaire du Cancer de Toulouse, Toulouse, France

**Keywords:** ROCK, melanoma, H1152, FasL, immune response

## Abstract

Ectopic Fas-ligand (FasL) expression in tumor cells is responsible for both tumor escape through tumor counterattack of Fas-positive infiltrating lymphocytes and tumor rejection though inflammatory and immune responses. We have previously shown that RhoA GTPase and its effector ROCK negatively control FasL membrane expression in murine melanoma B16F10 cells. In this study, we found that B16F10 treatment with the ROCK inhibitor H1152 reduced melanoma development *in vivo* through FasL membrane overexpression. Although H1152 treatment did not reduce tumor growth *in vitro*, pretreatment of tumor cells with this inhibitor delayed tumor appearance, and slowed tumor growth in C57BL/6 immunocompetent mice. Thanks to the use of mice-bearing mutated Fas receptors (B6/*lpr*), we found that reduced tumor growth, observed in immunocompetent mice, was linked to FasL overexpression induced by H1152 treatment. Tumor growth analysis in immunosuppressed NUDE and IFN-γ-KO mice highlighted major roles for T lymphocytes and IFN-γ in the H1152-induced tumor growth reduction. Histological analyses of subcutaneous tumors, obtained from untreated versus H1152-treated B16F10 cells, showed that H1152 pretreatment induced a strong intratumoral infiltration of leukocytes. Cytofluorometric analysis showed that among these leukocytes, the number of activated CD8 lymphocytes was increased. Moreover, their antibody-induced depletion highlighted their main responsibility in tumor growth reduction. Subcutaneous tumor growth was also reduced by repeated intravenous injections of a clinical ROCK inhibitor, Fasudil. Finally, H1152-induced ROCK inhibition also reduced pulmonary metastasis implantation independently of T cell-mediated immune response. Altogether, our data suggest that ROCK inhibitors could become interesting pharmacological molecules for melanoma immunotherapy.

## Introduction

Fas (also known as CD95/Apo-1) is a transmembrane protein belonging to the TNF receptor superfamily. It transmits apoptotic signaling in susceptible cells after being triggered by its natural ligand Fas-ligand (FasL) (CD95L/CD178) (1). The Fas receptor is ubiquitously expressed whereas FasL is mainly expressed in activated NK and T cells (2). Fas-mediated apoptosis is important in various biological processes including immune homeostasis through activation-induced cell death in T lymphocytes and cell-mediated cytotoxicity against tumor cells or following viral infection (3, 4). However, most cancer cells are relatively resistant to Fas-mediated apoptosis even tumor cells expressing high levels of Fas. Furthermore, it has recently been shown that Fas is required for the survival of cancer stem cells, and by a mechanism of retro-differentiation, it allows the emergence of new stem cells (5). FasL is also expressed in the eye and testis where its pro-apoptotic activity contributes to the immune privilege status of these tissues (6, 7).

Cancer progression is often associated with the acquisition of tumor cell immune resistance (8). FasL expression by tumor cells is one of the mechanisms responsible for this immunological escape. Indeed, its ectopic expression allows tumor cells to counterattack and induce apoptosis in Fas-expressing cytotoxic T lymphocytes and natural killer cells, thereby infiltrating the tumor microenvironment (9–12). However, although the Fas–FasL interaction is known to be important for human tumor progression, several opposite mechanistic roles are been clearly established (13). Some studies have reported that FasL tumor expression triggers a neutrophil-mediated inflammatory response and tumor rejection (14). However, it has also been shown that different FasL expression levels can modulate these effects. At high levels of expression, FasL was shown to trigger tumor rejection by a potent neutrophil-mediated local inflammation response, which initiates a T-lymphocyte-dependent anti-tumor-specific memory. In contrast, at low levels, FasL enhanced tumor growth by counterattacking anti-tumor effector lymphocytes (15).

Rho GTPases belong to the Ras superfamily of GTP-binding proteins (16). After activation, the RhoA GTPase interacts with intracellular target proteins or effectors to trigger a wide variety of cellular responses, including reorganization of the actin cytoskeleton, cell cycle progression, cells death, adhesion, metastasis, and gene transcription. One of its main effectors is ROCK kinases (Rho-associated protein kinases) (17). ROCK kinases are well known for modulating the actin cytoskeleton and actin–myosin contractility through the phosphorylation of the MYPT1 protein (18). In a previous study, we showed that RhoA GTPase and its effectors ROCK downregulate membrane FasL expression in B16F10 melanoma cells *in vitro*. We demonstrated that B16F10 cells overexpressing membrane FasL, thanks to pharmacological inhibition of the RhoA/ROCK pathway were able to induce the apoptosis of co-cultivated Fas-sensitive lymphocytes *in vitro* (19).

Many pharmacological molecules have been developed to target the RhoA/ROCK pathway. Statins inhibit the mevalonate pathway necessary for the prenylation and activation of GTPases. Some of them are widely prescribed as hypocholesterolemic agents and are now also being studied as potential anti-cancer agents (20). Targeting ROCK proteins has been shown to be useful in cardiovascular diseases, for example, the inhibitor Fasudil (HA 1077) is used to treat cerebral vasospasm (21) and it is intended in the treatment of pulmonary arterial hypertension (22). Moreover, due to their implication in migration and invasion capacities, RhoA/ROCK inhibitors are now being evaluated as anti-tumor therapies (23, 24).

In the present study, we have investigated the capacity of ROCK inhibitors, H1152 and Fasudil, to modulate FasL membrane expression in the B16F10 melanoma cell line and to control tumor growth *in vivo*. We demonstrate that ROCK inhibition with H1152 or Fasudil induces FasL overexpression at melanoma cell membranes *in vitro* and slows tumor growth *in vivo* by inhibiting melanoma cells invasion and drawing immune effector cells into the tumor microenvironment.

## Materials and Methods

### Tumor cell lines and animals

The murine melanoma cell line B16F10 and hybridomas against murine CD4 and murine CD8 were obtained from ATCC and were *in vitro* maintained by serial passages in RPMI 1640 medium (*Lonza*) supplemented with 10% FCS, 1 mM glutamine, and 1% penicillin–streptomycin–amphotericin B (*Lonza*). Cultures were tested monthly to ensure that they were mycoplasm-free. About 6- to 9-week-old female C57BL/6 wt and NMRI nude mice were obtained from *Elevages Janvier*. C57BL/6 IFN-γ-KO mice were kindly provided by Pr. Jean-Charles Guéry (INSERM U1043, Toulouse) and B6/*lpr* mice were kindly provided by Pr. Pierre Bobé (CNRS UMR7592, Paris). The experiments in mice have been done in the appropriate conditions of husbandry, experimentation, and care, controlled by the Ethic Comity of the Institut Claudius Regaud under the control of the Regional Comity of Midi-Pyrénées (France). Our protocols were validated and received the agreement number ICR-2009-0011.

### Treatment of melanoma cells

Melanoma cells were treated *in vitro* with two ROCK inhibitors: H1152 (*Calbiochem*) at 1 μM for 24 h and Fasudil (Selleckchem) for 24 h at indicated concentrations from 5 to 25 μM. Fasudil was also injected intravenously (25 mg/kg) every 2 days for 13 days in mice-bearing subcutaneous B16F10 tumors.

### Flow cytometry analyses

FITC-conjugated anti-Thy1.1, PE-conjugated anti-FasL, PE-conjugated anti-CD69, PE-conjugated anti-CD4, APC-conjugated anti-CD8 mAbs, and PE-conjugated anti-CD107a, corresponding isotype controls and 7-aminoactinomycin D (7-AAD) were purchased from *BD Biosciences*. After 30 min incubation, the stained cells were analyzed on a BD FACS Calibur (*Becton Dickinson*) and results were analyzed with FlowJo software. Results are illustrated as percentage of positive cells for each molecule.

### *In Vitro* proliferation

1 × 10^5^ B16F10 cells, either untreated or pretreated for 24 h with 1 μM of H1152, were cultivated *in vitro*. B16F10 cells were counted after 2, 4, 6, and 8 days of culture with Cell Counter (*Coulter*) to evaluate their *in vitro* proliferation, which allows evaluating the toxicity of the H1152 treatment.

### Subcutaneous tumor growth

To study the tumor growth, all mice were injected subcutaneously with 3 × 10^5^ B16F10 cells either untreated or pretreated with 1 μM of H1152 for 24 h. Melanoma cells were washed twice in PBS before injection. Moreover, to study tumor growth with *in vivo* Fasudil injection, all mice were injected subcutaneously with 3 × 10^5^ untreated B16F10 cells and then treated with intravenous injections of Fasudil (25 mg/kg) or PBS every 2 days for 13 days. Animals were monitored for tumor growth every 2–3 days by palpation and diameters of the tumors were measured using a Vernier caliper. Tumor-bearing animals were sacrificed at day 14 after tumor injection. Results are expressed as mean surface ± SD (error bars, *n* = 13–16 mice).

### CD4 and CD8 cells depletion in C57BL/6 wt mice

Neutralizing antibodies against murine CD4 and CD8 are produced from hybridomas TIB-207 and TIB-105, respectively. Antibodies were isolated and purified in our laboratory by affinity chromatography with ÄKTA Purifier system (GE Healthcare Life Sciences).

To validate their *in vivo* efficiency, these antibodies were injected intraperitoneally in C57BL/6 wt mice daily for three consecutives days at 200 μg for each mouse. On day 4, lymph nodes and spleen of each mouse were recovered and crashed in a manual manner through a Cell Strainer (*Falcon*). Then, extracted cells were analyzed for CD4+ and CD8+ population by flow cytometry.

For tumor growth experiments, anti-CD4 and anti-CD8 neutralizing antibodies were injected intraperitoneally in C57BL/6 wt mice at day 0, 1, 2, 4, 7, and 11 after tumor inoculation at 200 μg for each mouse.

### Tumor-infiltrating lymphocytes analyzes

1 × 10^6^ B16F10 cells, untreated or pretreated 24 h with 1 μM H1152, were injected subcutaneously in the flanks of C57BL/6 wt mice. Four days later, tumor masses were recovered and dissociated with the GentleMACS Dissociator (*Miltenyi*) according to manufacturer’s instructions. Quantification by flow cytometry of lymphocytes was performed thanks to the staining of extracted cells with anti-CD8, anti-Thy1.1, and anti-CD69 antibodies and also a cell viability marker 7-AAD. Similarly, CD107a-positive lymphocytes were quantified among CD8 T lymphocytes in the tumor masses 7 days after B16F10 cells injection.

### Cell migration *In Vitro* assays

*In vitro* migration studies were performed using triplicate or quadruplicate wells. Migration assays were performed with 8-μm pore size transwell system (BD Biosciences).

B16F10 cells were untreated or pretreated 24 h with 1 μM H1152. Then, 2.5 × 10^4^/well melanoma cells were added in RPMI 1640 + 2% FCS in the upper compartment of the filter. The bottom chamber was filled with RPMI 1640 + 10% FCS. After 24 h, cells on the bottom surface of the filter were stained and counted. Photos were taken with an Eclipse Ti microscope (Nikon Instruments) and a CoolSNAP HQ2 camera (Photometrics) in three randomized fields.

### Histology

Mice tissues were taken from the area surrounding the B16F10 cells inoculation sites and fixed in formol. Tissues were then embedded in paraffin wax and 5-μm serial sections were taken. Sections were then stained with hematoxylin and eosin (H&E) to estimate the tumor mass and infiltrate.

### Pulmonary metastases implantation

To study pulmonary metastases implantation, C57BL/6 wt and NMRI nude mice were injected intravenously (i.v.) with 2 × 10^5^ B16F10 cells either untreated or pretreated 24 h with 1 μM H1152. The melanoma cells were washed twice in PBS before injection. Mice were sacrificed 12 days later. Macroscopic metastases were detected visually and double blind quantified. Then, lungs were fixed in formalin and paraffin embedded to visualize microscopic metastases. Photos were taken with a DMR microscope (Leica Microsystems) and a DS-Fi1 camera (Nikon Instruments). Results are expressed as mean ± SD (error bars, *n* = 12 mice). The experiments included four mice per group and were repeated twice.

### Statistical analysis

Statistical analyses were performed using GraphPad Prism software. Significance of analyses was assessed by *t*-test or Tukey one-way or two-way ANOVA test. All statistic tests were two sides. The values are expressed as means ± SD in the figures. *P*-values <0.05 were considered statistically significant.

## Results

### H1152 treatment of B16F10 melanoma cells *In Vitro* induces FasL membrane overexpression without affecting proliferation

**Figure 1:**
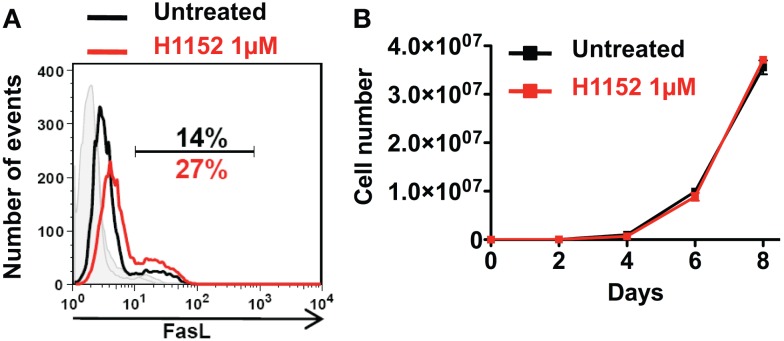
**Inhibition of ROCK increases FasL membrane expression on B16F10 melanoma cells without cell toxicity**. B16F10 cells were treated or not with 1 μM of H1152 for 24 h then membrane FasL expression was quantified using flow cytometry **(A)**. B16F10 cells were treated or not with 1 μM of H1152, then these cells were put in culture and every 2 days pretreated or untreated B16F10 cells were counted to evaluate the *in vitro* proliferation **(B)**. Results are expressed as mean ± SD (error bars, *n* = 3 experiments).

In a previous study, we showed that RhoA/ROCK pathway inhibition induced the overexpression of membrane FasL in B16F10 melanoma cell line. And in particular, inhibition of effectors of RhoA, the ROCK kinases, with the pharmacological H1152 inhibitor at 0.5 μM for 24 h induced overexpression of membrane FasL in B16F10 cells (19). Here, we confirm this since inhibition of ROCK kinases, with H1152 at 1 μM for 24 h also induced overexpression of membrane FasL in B16F10 cells (Figure 1A). To verify that this H1152 treatment was not toxic for B16F10 cells, cells were untreated or treated for 24 h with 1 μM H1152, then cultivated *in vitro*, and counted every 2 days. H1152 by itself was not toxic since B16F10 cells proliferated *in vitro* at similar rates after treatment (Figure 1B). These data show that 1 μM of H1152 is not toxic for these melanoma cells and that membrane FasL overexpression does not interfere with B16F10 cell *in vitro* proliferation.

### H1152 pretreatment reduces local B16F10 melanoma growth *In Vivo* in a Fas/FasL pathway-dependent manner

**Figure 2:**
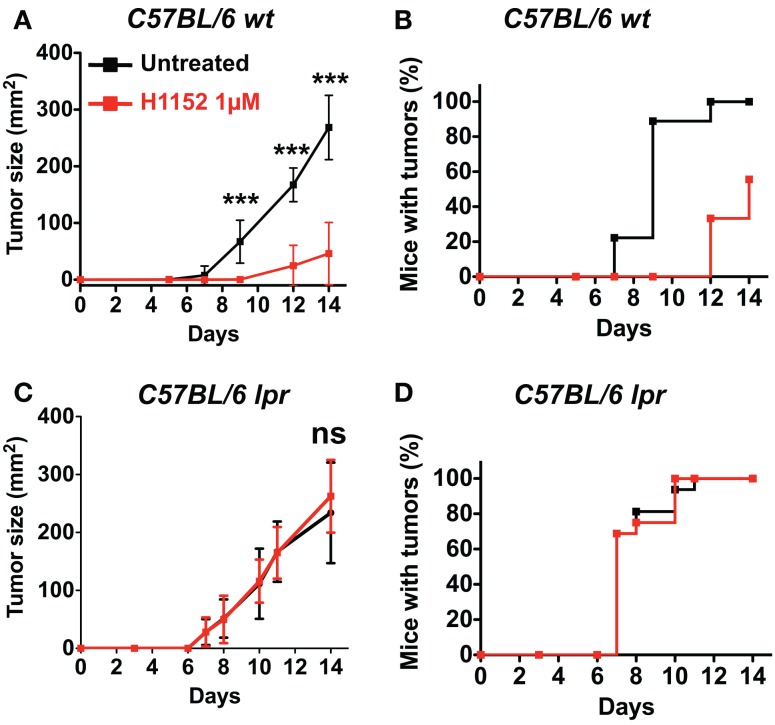
**Inhibition of ROCK reduces *in vivo* melanoma growth through Fas/FasL pathway**. 3 × 10^5^ B16F10 cells pretreated or not with 1 μM H1152 for 24 h were injected subcutaneously in C57BL/6 immunocompetent mice (*n* = 20 mice for each group). *In vivo* tumor growth was monitored regularly **(A)** and number of tumor-free mice was also assessed **(B)**. Same experiments were performed in Fas-deficient C57BL/6 *lpr* mice **(C,D)** (*n* = 16 mice for each group). Results are expressed as mean ± SD. ***P < 0.001 versus control using the Tukey ANOVA test.

Based on previous studies reporting an ambiguous role for FasL in tumor development (14, 25), we wondered whether H1152-induced FasL membrane overexpression would modulate tumor growth *in vivo*. We performed subcutaneous injection of 3 × 10^5^ B16F10 cells, either untreated or pretreated with 1 μM H1152 for 24 h, into the flank of C57BL/6 wild-type mice. The resulting tumors showed that B16F10 cells pretreated with H1152 grew significantly slower than untreated cells (Figure 2A). In addition, the appearance of tumors in mice was delayed by 5 days (Figure 2B). We then investigated whether the B16F10 melanoma deceleration observed in immunocompetent mice was dependent on FasL overexpression. For this purpose, we used C57BL/6 mice naturally carrying the *lpr* mutation, which leads to a truncated and inactive form of the Fas receptor (B6/*lpr* mice) (26). In these mice, we performed the same subcutaneous injections of 3 × 10^5^ B16F10 cells, either untreated or pretreated with 1 μM H1152 for 24 h. Unlike our observations in immunocompetent mice, we did not observe either a reduction in tumor growth rate (Figure 2C) or a delay in tumor appearance (Figure 2D). These results confirm an essential role for membrane FasL overexpression in B16F10 cells in reducing tumor growth *in vivo* after pretreatment with H1152.

### Slowing down of melanoma growth after H1152 pretreatment is dependent on the IFN-γ-mediated T cell immune response

**Figure 3:**
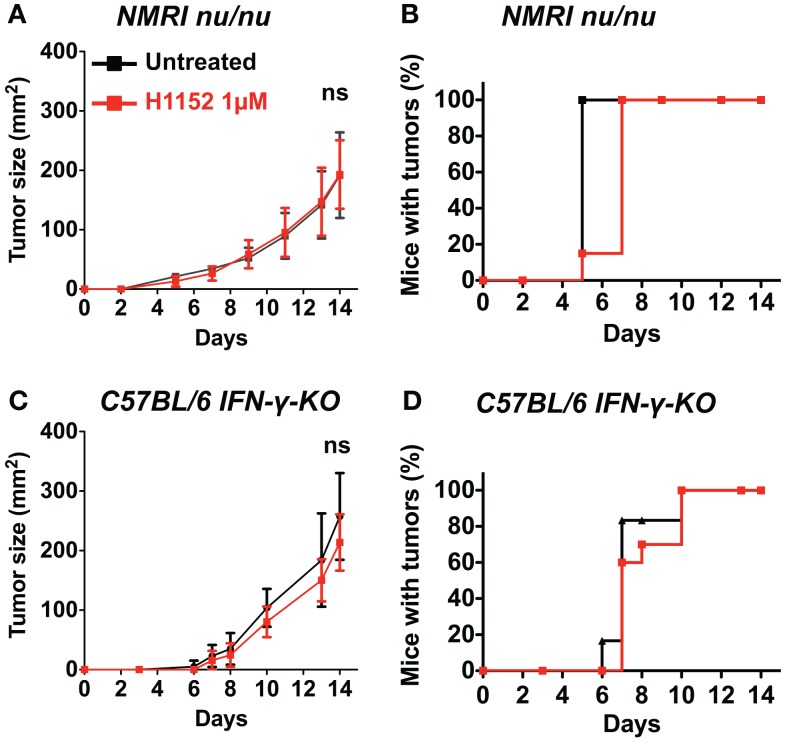
**An IFN-γ-dependent lymphocytes response is involved in the *in vivo* melanoma shrinkage observed in C57BL/6 immunocompetent mice following ROCK inhibition**. 3 × 10^5^ B16F10 cells pretreated or not with 1 μM of H1152 for 24 h were injected subcutaneously in nude NMRI mice (*n* = 12 mice for each group). *In vivo* tumor growth was monitored regularly **(A)** and number of tumor-free mice was also assessed **(B)**. Same experiments were performed in IFN-γ-KO C57BL/6 mice **(C,D)** (*n* = 16 mice for each group). Results are expressed as mean ± SD. ns versus control using the Tukey ANOVA test.

To further investigate the immune mechanisms implicated in FasL-mediated B16F10 melanoma slower growth *in vivo*, 3 × 10^5^ B16F10 cells were pretreated or not with 1 μM H1152 for 24 h then subcutaneously injected into the flank of IFN-γ-KO C57BL/6 mice and NMRI nude immunosuppressed mice. Tumor growth rate monitoring revealed no reduction in NMRI nude mice (Figure 3A) or IFN-γ-KO C57BL/6 mice (Figure 3C) when B16F10 cells were pretreated with H1152 compared to controls. No delay in tumor appearance was observed in the same mice (Figures 3B,D). These results strongly implicate a role for the adaptive immune response in the reduction of H1152-induced FasL-over-expressing B16F10 tumor growth through an IFN-γ-dependent mechanism.

### Pretreatment with H1152 induces a massive leukocytes infiltration into the tumor site, including activated TCD8+ lymphocytes

**Figure 4:**
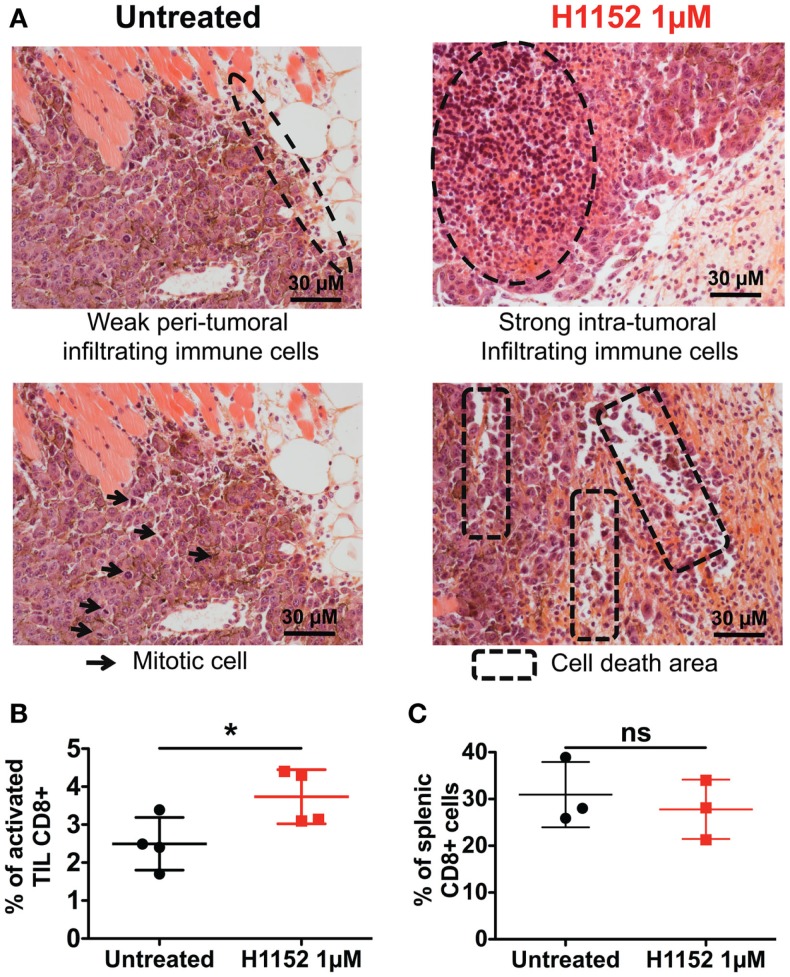
**H1152 pretreatment recruits a massive infiltration of immune cells into the tumor site**. B16F10 cells pretreated or not with 1 μM of H1152 for 24 h were injected subcutaneously in C57BL/6 mice. Four days later, mice were killed and tumors were collected for histology. Hematoxylin and eosin-stained 5-μm paraffin-mounted sections were generated **(A)**. Also, tumor masses were recovered and tumor-infiltrating cells were extracted with the Gentle MACS Dissociator according to manufacturer’s instructions, and TIL Thy1.1+CD8+CD69+7-AAD−were analyzed by flow cytometry **(B)**. In the same mice, spleens were recovered, splenic cells were extracted by manual dissociation through a Cell Strainer, and CD8+ cells were analyzed by flow cytometry **(C)**. Results are expressed as mean ± SD. *P < 0.05 versus control using the Student *t*-test **(B,C)**.

Since the immunological status of mice appeared to be essential for observing the biological effects of ROCK inhibition-induced FasL overexpression on B16F10 tumor growth, we next performed histological and flow cytometric analyses to visualize any modifications of the immune microenvironment in our model. First, double-blind analysis of tumoral and surrounding tissues stained by hematoxylin and eosin (H&E) was carried out. To prepare these samples, 1 × 10^6^ B16F10 cells pretreated or not with 1 μM H1152 for 24 h were injected subcutaneously into C57BL/6 mice. Four days after tumor inoculation, tumors were recovered and embedded in paraffin. Examination of the H&E stained tumor sections revealed that H1152 pretreatment induced a massive infiltration of leukocytes into the tumors whereas injection with control cells led to a weak infiltration with leukocytes located at the tumor periphery (Figure 4A). These leukocytes could be monocytes, lymphocytes, or granulocytes, mainly neutrophils, as previously described (27). In addition, in contrast to control cells, pretreated tumors showed a lower mitotic index, reduced cohesion, and the presence of large cell death areas (Figure 4A). Flow cytometry analysis showed the infiltration of CD8+ lymphocytes in these tumors. In a separate experiment, 4 days after tumor inoculation under the same conditions, tumors were recovered, dissociated with a GentleMACS dissociator, and cells were then analyzed by flow cytometry. Results showed a stronger infiltration of activated CD8+ T lymphocytes (CD3+ CD8+ CD69+ cells) in pretreated tumors compared to control tumors (Figure 4B). We also checked that this tumoral infiltration was not due to a global higher amount of CD8+ T lymphocytes present in these mice (as shown in Figure 4C). To know whether these infiltrating lymphocytes have cytotoxic capacities, in another separate experiment, 7 days after tumor inoculation under the same conditions, tumors were recovered, dissociated with a GentleMACS dissociator, and membrane CD107a expression was analyzed by flow cytometry on CD8 T lymphocytes. Results showed a significant increase in the infiltration of cytotoxic CD107a+ lymphocytes in pretreated tumors compared to control tumors (Figure S1 in Supplementary Material).

### Depletion of CD8+ and CD4+ cells limits the *In Vivo* melanoma slowing down observed after H1152 pretreatment

**Figure 5:**
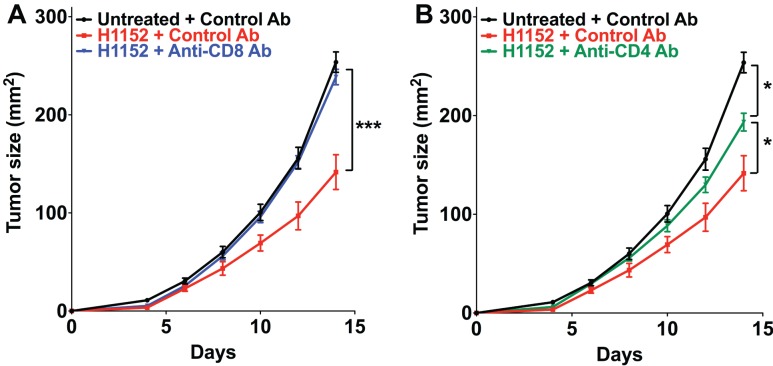
**Infiltration of T CD8+ cells in tumors is mainly responsible for B16F10 tumor growth slowing down and TCD4+ cells are lightly involved**. B16F10 cells pretreated or not with 1 μM of H1152 for 24 h were injected subcutaneously in C57BL/6 mice (13 < *n* < 22 mice for each group). Mice received five intraperitoneal injections of anti-CD8 **(A)** or anti-CD4 **(B)** neutralizing antibody or control antibody at 200 μg between day 0 and day 11 after tumor injection. Then, *in vivo* tumor growth was monitored regularly until sacrifice at day 14. Results are expressed as mean ± SEM. *P < 0.05; ***P < 0.001 versus control using the Tukey ANOVA test **(A,B)**.

The above results in immunocompetent mice showed that the control of tumor cells growth following ROCK inhibition-induced FasL overexpression occurs by the establishment of an adaptive immune response that is mainly managed by TCD8+ and TCD4+ cells. We therefore investigated the role of these immune populations in our model by specifically depleting C57BL/6 mice of CD8+ or CD4+ cells using the neutralizing antibodies TIB-105 and TIB-207, respectively, which are derived from hybridoma. Either neutralizing antibodies or control antibodies were injected intraperitoneally into mice at 200 μg per injection on days 0, 1, 2, 4, 7, and 11 after tumor inoculation. On day 0, 3 × 10^5^ B16F10 cells, pretreated or not with 1 μM H1152 for 24 h, were subcutaneously injected into these mice and then tumor growth was monitored. The *in vivo* efficiency of the TIB-105 and TIB-207 neutralizing antibodies on depleting the CD8+ and CD4+ cell populations in our conditions was assessed 4 days after injection. Results confirmed that these antibodies efficiently depleted their respective cell populations in the spleen and lymph nodes (Figures S2A–D in Supplementary Material). As initially observed, B16F10 cells pretreated with H1152 had a slower *in vivo* growth compared to control cells (Figure 5A). Interestingly, depletion of the CD8+ population completely abolished this reduction in the rate of tumor growth (Figure 5A). Depletion of the CD4+ cell population had an intermediate effect on H1152-induced melanoma slowing down since it did not completely restore tumor growth to that of the control conditions (Figure 5B). Thus, these results show an important role for CD8+ lymphocytes in the control of H1152-pretreated tumor growth and a lesser role for CD4+ cells.

### Fasudil intravenous injections reduce local B16F10 melanoma growth *In Vivo*

**Figure 6:**
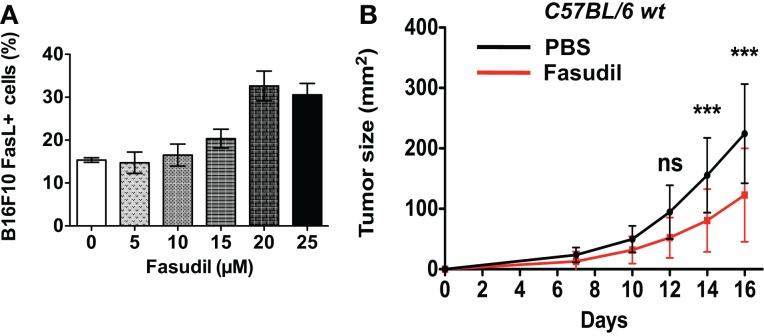
**Fasudil treatment induces membrane FasL overexpression on B16F10 cells and reduces tumor growth after intravenous administration in mice**. B16F10 cells were treated or not with Fasudil at indicated concentrations for 24 h and then membrane FasL expression was quantified using flow cytometry **(A)**. 3 × 10^5^ B16F10 cells were subcutaneously injected in C57BL/6 mice and then mice were injected intravenously with Fasudil (25 mg/kg) or PBS every 2 days for 13 days (*n* = 13 mice for each group) **(B)**. Results are expressed as mean ± SD. ***P < 0.001 versus control using the Tukey ANOVA test.

We next analyzed the impact of repeated intravenous injections with the clinically used ROCK inhibitor, Fasudil (HA 1077) on B16F10 melanoma growth *in vivo*. We first performed a dose–response analysis of *in vitro* treatment with Fasudil on membrane FasL expression in B16F10 cells. Results showed a Fasudil-induced overexpression of membrane FasL at 20 and 25 μM (Figure 6A). Then 3 × 10^5^ untreated B16F10 cells were subcutaneously injected into the flank of C57BL/6 wild-type mice, which were then treated with intravenous injections of Fasudil (25 mg/kg) or PBS every 2 days for 13 days. The resulting tumors showed that B16F10 tumors grew significantly slower in Fasudil-injected mice than in control mice, confirming the ROCK inhibitors capacity to reduce melanoma growth *in vivo* (Figure 6B). As FasL, used systemically, induce apoptosis in hepatocytes, we controlled that no damages were detectable in the liver of Fasudil-injected mice.

### Pretreatment with H1152 inhibits *In Vitro* migration and reduces pulmonary metastasis implantation of B16F10 cells

**Figure 7:**
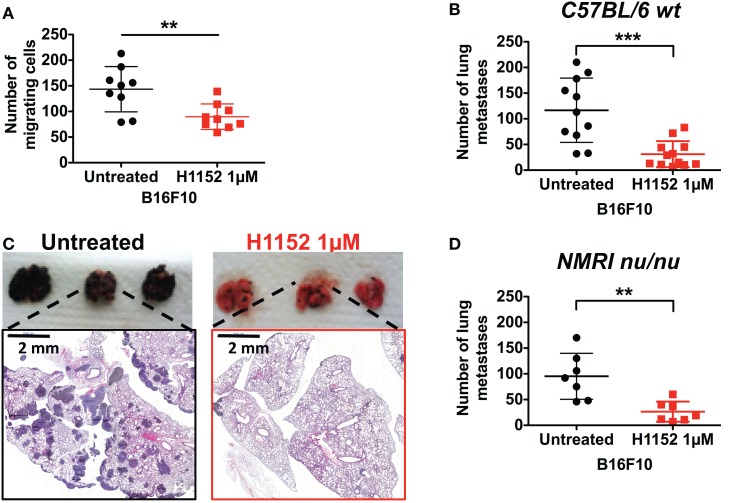
**ROCK inhibition with H1152 reduces *in vitro* cell migration and *in vivo* metastases’ establishment in lung without implication of T lymphocytes**. *In vitro* migration of B16F10 cells untreated or pretreated with H1152 1 μM for 24 h was analyzed using transwell assays **(A)**. B16F10 cells untreated or pretreated with H1152 1 μM for 24 h were injected intravenously in C57BL/6 mice. Twelve days after inoculation, lungs were recovered and macroscopic pulmonary metastases were quantified **(B)**. Lung photomicrographs are shown for representative lungs of C57BL/6 mice **(C)**. Results are expressed as mean ± SD. **P < 0.005; ***P < 0.001 versus control using the Student’s *t*-test. B16F10 cells untreated or pretreated with H1152 1 μM for 24 h were injected intravenously in nude NMRI mice **(D)**. Twelve days after inoculation, lungs were recovered and macroscopic pulmonary metastases were quantified. Results are expressed as mean ± SD. **P < 0.005 versus control using the Student’s *t*-test.

In melanoma pathology, metastasis is the most dangerous clinical step and current therapies have limited efficiency at this stage. Moreover, ROCK kinases are well recognized as regulators of cell migration and cell invasion through their modulation of the actin cytoskeleton (18, 24). Therefore, we analyzed the impact of H1152 treatment on B16F10 cell motility and invasion. First, *in vitro* transwell assays were used to show that H1152 pretreatment (1 μM for 24 h) inhibited the migration of B16F10 cells (Figure 7A). Then, B16F10 cells either untreated or pretreated with H1152 were injected intravenously into the tail vein of C57BL/6 wild-type mice and lungs were recovered 12 days later. Macroquantification revealed a lower number of metastases in the lungs of mice injected with H1152-pretreated B16F10 cells compared to control cells (Figure 7B), showing that H1152 pretreatment decreased the metastatic implantation capacity of melanoma cells. Figure 7C illustrates the lung H&E staining of a representative experiment allowing microquantification of lung metastases. We wondered whether this reduction in metastasis implantation was dependent on an adaptive immune response. Therefore, the same experiments were performed in NMRI nude mice. The number of lung metastases was decreased in NMRI nude mice injected with H1152-pretreated B16F10 cells versus untreated cells. Moreover, the ratio between the number of metastases obtained with untreated versus H1152-pretreated B16F10 cells (3.7-fold) was similar in immunocompetent and immunocompromised mice (Figure 7D), strongly suggesting that T lymphocytes were not involved in the H1152-dependent reduction of metastasis. Therefore, we concluded that this decrease in metastatic potential was intrinsically linked to ROCK inhibition.

## Discussion

Melanomas are immunogenic tumors, which express tumor antigens and other molecules that are recognized by the effectors of the innate and adaptive immune responses. This recognition can be avoided via many mechanisms leading to the immune escape of tumors. One of these mechanisms is the ectopic expression of FasL on tumor cell membranes that triggers a counterattack against Fas-expressing lymphocytes (13, 28). However, this FasL-mediated tumor counterattack can be reversed as FasL overexpression in cancer cells is also known to elicit anti-tumor effects (14, 15, 29).

The aim of this study was to find out whether treatment with ROCK inhibitors, such as H1152 and Fasudil, could induce a melanoma overexpression of FasL capable of promoting tumor rejection. If this was the case, such inhibitors might therefore be of interest for the treatment of metastatic melanoma. Recently, a study reported that the ambivalent role of FasL in cancer could be related to the timing of its expression. In fact, they showed that when FasL was initially expressed in injected cancer cells, it elicited anti-tumor activity, but when FasL expression was delayed after tumor implantation, the tumor microenvironment abrogated the FasL-mediated anti-tumor activity (27). Moreover, a separate previous publication suggested that the ambivalent role of FasL in melanoma is instead connected to its level of expression, with a high expression favoring tumor rejection and a low expression inducing tumor escape through FasL counterattack (15).

Altogether, the experiments presented here show that treatment of B16F10 melanoma cells with H1152 induces FasL membrane overexpression without interfering with proliferation *in vitro*. Moreover, the clinically used ROCK inhibitor, Fasudil, is also able to reduce melanoma growth by intravenous injections. Previously, another ROCK inhibitor (Y27623) was used *in vivo* and it inhibited melanoma growth when it was injected intraperitoneally (30). In our experiments, the ROCK inhibition-induced FasL overexpression triggers a protective immune tumor-microenvironment *in vivo*. Indeed, we show that tumor development from injected H1152-treated B16F10 cells was significantly reduced *in vivo*. This effect is dependent on the Fas/FasL pathway and is mainly mediated by the immune response of IFN-γ-T CD8+ lymphocytes. Activated macrophages could also be involved as previously shown in mice-bearing intraocular tumors (31) or infected by *Leishmania major* (32). In these mice, a synergy between Fas–FasL pathway and IFN-γ was necessary to eliminate the tumors or for resolution of parasite-induced lesions by activated macrophages. Our results are consistent with studies reporting that FasL overexpression is an inducer of anti-tumor immune responses (15, 27, 29, 33). However, FasL has already been involved in CD8T-cell infiltration into tumors, but contrary to what we describe here with melanoma cells overexpressing FasL in membrane, FasL expression on endothelial cells causes reduced CD8T-cell infiltration into the tumor (34).

Tumor-infiltrating CD8 lymphocytes play a major role in the reduction of growth of B16F10 cells over-expressing FasL. Indeed, activated CD8 lymphocytes mainly infiltrate these tumors and were responsible for the reduction in the rate of tumor growth, since the specific *in vivo* depletion of the CD8+ population restored tumor growth from H1152-pretreated B16F10 cells even when FasL was overexpressed in these cells. On the other hand, the specific depletion of CD4+ cells had an intermediate effect and partially restored tumor growth. This intermediate effect could be explained by the diversity and opposing effects of the CD4+ T cell subsets present in the tumor microenvironment, including anti-tumoral Th1 cells and immunosuppressive T regulatory (Treg) cells (35). Using B16F10 cells transfected with FasL, it has previously been shown that Treg cells limit the inflammatory response by inhibiting neutrophils accumulation and survival, thereby favoring melanoma growth (36). Here, we generated FasL overexpression through ROCK inhibition, and our depletion experiments show that the selective elimination of CD8+ cells is sufficient to restore normal tumor growth.

We also demonstrate here that H1152-induced slowing down of melanoma growth is associated with a massive infiltration of leukocytes. Our flow cytometric analyses showed that the number of activated CD8+ lymphocytes is increased in these leukocytes. These results are consistent with those of Erdag et al. who demonstrated that higher densities of CD8+ T cells correlate with a better survival in melanoma patients (37). Moreover, studies led by Prof. Galon’s team have linked a good survival prognosis for colorectal cancer patients with infiltration of T CD8+ CD45RO+ memory cells (38, 39). Memory immunity has not been evaluated in our present study but we can speculate that the H1152-induced increase in tumor-infiltrating activated CD8+ lymphocytes could generate CD8+ CD45RO+ memory cells.

Our final results show that ROCK inhibition reduces cell migration and pulmonary metastasis implantation. These effects are mainly due to the intrinsic capacity of the H1152 ROCK inhibitor to reduce ROCK kinase-mediated control of cell migration and cancer cell invasion (18). These observations were reinforced by our experiments carried out in immunosuppressed NMRI nude mice, which revealed that an adaptive immune response involving T lymphocytes was not responsible for the reduction in metastasis. In agreement with this, some studies have now recognized FasL as an inducer of cell invasion and cell migration in addition to its well-established role in inducing apoptosis (40). However, it is well known that NK cells play an important role in the control of metastatic processes (41), so since the mutation present in NUDE mice does not affect NK cell generation and activity, a ROCK inhibition-induced innate immunity-dependent effect on metastasis development cannot yet be excluded.

In conclusion, our results show that Rho-kinase inhibitors, H1152 and Fasudil, decrease melanoma growth, and pretreatment of B16F10 melanoma cells with H1152 inhibitor promotes an anti-tumor immune response through increased FasL expression. Therefore, these inhibitors could become interesting pharmacological molecules for melanoma immunotherapy.

## Conflict of Interest Statement

The authors declare that the research was conducted in the absence of any commercial or financial relationships that could be construed as a potential conflict of interest.

## Supplementary Material

The Supplementary Material for this article can be found online at http://journal.frontiersin.org/article/10.3389/fonc.2015.00156
