# Supplementary material for: In vivo Effects in Melanoma of ROCK Inhibition-Induced FasL Overexpression
Source: Front Oncol. 2015 Jul 14;5:156. doi: 10.3389/fonc.2015.00156 (PMC4500923; doi:10.3389/fonc.2015.00156)
Supplement: Supplementary file 1 [file Presentation_1.PDF]

Supplementary Material

In vivo effects in melanoma of ROCK inhibition-induced FasL overexpression

Teiti Iotefa[1,2], Bertrand Florie[1,2], Pich Christine[1,2], Gence Rémi[1,2], Lajoie-Mazenc Isabelle[1,2], Rochaix Philippe[2,3], Favre Gilles[1,2,3] and Tilkin-Mariamé Anne-Françoise[1,2*].

1 INSERM UMR 1037, CRCT, Team 3 RhoGTPases in tumor progression, Toulouse, France
2 Université Paul Sabatier, Toulouse, France
3 Institut Universitaire du Cancer de Toulouse, Toulouse, France

***Corresponding author**: Tilkin-Mariamé Anne-Françoise[1,2] CRCT, INSERM U1037 - Université Toulouse 3 - ERL5294 CNRS, 2 avenue Hubert Curien, 31037 TOULOUSE - FRANCE
E-mail: anne-francoise.tilkin@inserm.fr

Figure S1

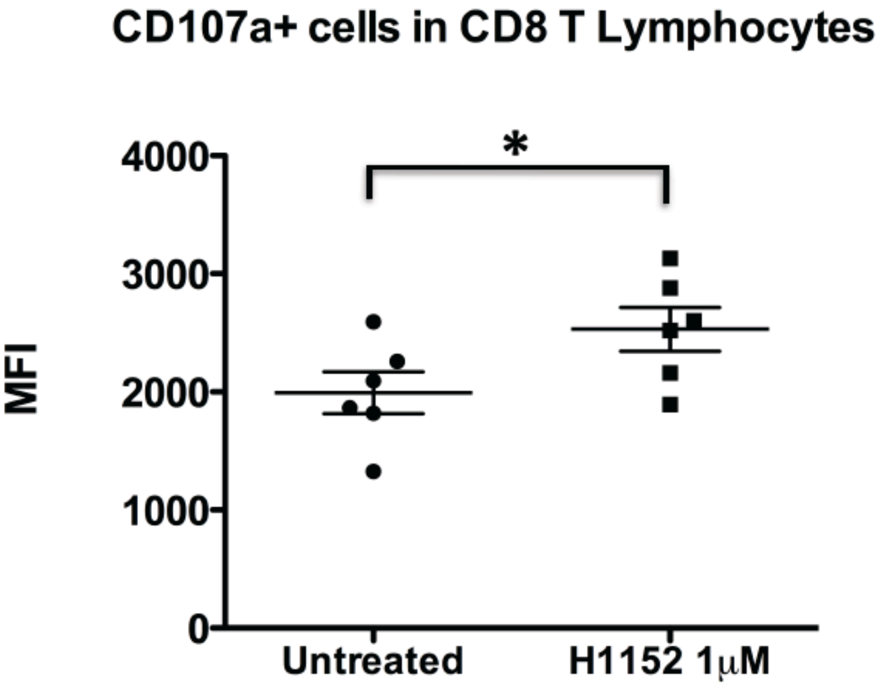

Supplemental Figure 1. H1152 pretreatment recruits infiltration of cytotoxic CD8 T lymphocytes into the tumor site.
B16F10 cells pretreated or not with 1 µM of H1152 for 24 h were injected subcutaneoulsy in C57BL/6 mice. Seven days later, mice were killed and tumors were collected and tumor-infiltrating cells were extracted with the Gentle MACS Dissociator according manufacturer's

instructions and CD8 T lymphocytes expressing membrane CD107a+ were analyzed by flow cytometry. Results are expressed as mean ± SD. *P < 0.05 versus control using the Student t-test.

Figure S2

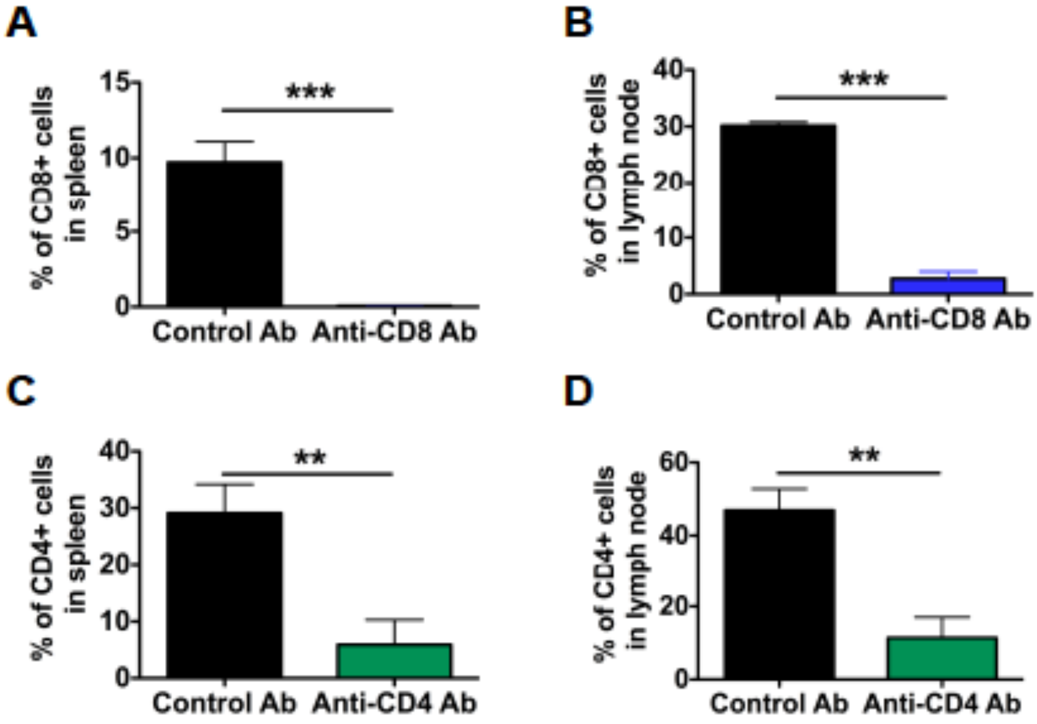

Supplementary Figure 2. Neutralizing antibodies against CD8+ and CD4+ cells are functional in mice.

C57BL/6 mice were injected with anti-CD8 or anti-CD4 neutralizing antibody or control antibody intraperitoneally at 200 µg each day for three consecutive days. The fourth day, spleens (A, C) and lymph nodes (B, D) were recovered, cells were extracted by manual dissociation through a Cell Strainer and CD8+ (A, B) and CD4+ (C, D) cells were analysed by flow cytometry. **P < 0.005; ***P < 0.001 versus control using the Student t-test.
